# Systematic review of interventions to increase the delivery of preventive care by primary care nurses and allied health clinicians

**DOI:** 10.1186/s13012-016-0409-3

**Published:** 2016-04-06

**Authors:** Kathleen M. McElwaine, Megan Freund, Elizabeth M. Campbell, Kate M. Bartlem, Paula M. Wye, John H. Wiggers

**Affiliations:** 1 Population Health, Hunter New England Local Health District, Booth Building, Wallsend Health Services, Longworth Avenue, Wallsend, NSW 2287 Australia; 2 Faculty of Health, The University of Newcastle, University Drive, Callaghan, NSW 2308 Australia; 3 Hunter Medical Research Institute, Clinical Research Centre, Lot 1 Kookaburra Circuit, New Lambton Heights, NSW 2305 Australia; 4 School of Psychology, Faculty of Science and Information Technology, The University of Newcastle, University Drive, Callaghan, NSW 2308 Australia; 5 Postal address: Locked Bag 10, Wallsend, NSW 2287 Australia

**Keywords:** Prevention and control, Primary health care, Review, Systematic

## Abstract

**Background:**

Primary care nurses and allied health clinicians are potential providers of opportunistic preventive care. This systematic review aimed to summarise evidence for the effectiveness of practice change interventions in increasing nurse or allied health professional provision of any of five preventive care elements (ask, assess, advise, assist, and/or arrange) for any of four behavioural risks (smoking, inadequate nutrition, alcohol overconsumption, physical inactivity) within a primary care setting.

**Methods:**

A search of Medline, Embase, PsycInfo, and CINAHL databases was undertaken to locate controlled intervention trials published between 1992 and May 2014 that provided practice change interventions to primary care nurses and/or allied health professionals to increase preventive care. The effect of interventions aimed at increasing the provision of any of the five care elements for any of the four behavioural risks was examined. A narrative synthesis was utilised.

**Results:**

From 8109 articles, seven trials met the inclusion criteria. All trials bar one, assessed multi-strategic practice change interventions (three to five strategies) focused on care by nurses (six trials) or mixed nursing/allied health clinicians. One trial examined care provision for all four risks, five trials examined care for smoking only, and one trial examined care for alcohol consumption only. For the six trials reporting significance testing (excludes one smoking care trial), significant effects favouring the intervention group were reported in at least one trial for smoking risk assessment (2/4 trials reported an effect for at least one analysis of an assessment outcome), brief advice (2/3), assistance (2/2), and arranging referral (2/3); alcohol risk assessment (1/2) and brief advice (1/2); inadequate nutrition risk assessment (1/1); and physical inactivity risk assessment and brief advice (1/1). When the number of analyses undertaken within trials focusing on smoking care was considered, the results were less promising (e.g. of the 15 analyses conducted on brief advice variables across three trials, four showed a positive effect).

**Conclusions:**

Evidence for the effect of practice change interventions on preventive care by primary care nurses or allied health providers is inconclusive given the small number of trials and inconsistency of results between and within trials.

**Systematic review registration number:**

None

**Electronic supplementary material:**

The online version of this article (doi:10.1186/s13012-016-0409-3) contains supplementary material, which is available to authorized users.

## Background

The routine delivery of primary preventive care by primary health care providers is recommended by national and international clinical guidelines [1–8] to reduce the disease burden caused by four priority risk behaviours: tobacco smoking, inadequate nutrition, alcohol overconsumption, and physical inactivity [9–11]. Primary health care clinicians encompass a variety of health care professionals such as nurses and allied health professionals including physiotherapists, dieticians, and occupational therapists, among others [12–16].

Such guidelines recommend that preventive care is provided by a range of primary health care providers including nurses (e.g. practice nurses, nurses, midwives) [1–3, 5] and allied health clinicians [5, 7] (e.g. health visitors, physiotherapists, exercise professionals, and health trainers) [5]. Preventive care is recommended to be provided for multiple risks [1, 2], routinely and opportunistically when clients present for reasons not necessarily related to their preventable health risk behaviours [1–8]. Five preventive care elements are recommended to meet this guidance: *asking* all patients about the four behavioural risks (risk assessment); *assessment* of readiness to change and dependence (for smoking and alcohol); brief, non-judgemental *advice* with patient educational materials and motivational interviewing; *assistance* by providing motivational counselling and pharmacotherapy if required (for nicotine or alcohol dependence); and *arrangement* of a referral to telephone support services, group lifestyle programs, or an individual provider (e.g. dietician), and a follow-up visit where applicable [17, 18]. These elements are commonly referred to as the 5A’s [1].

Cochrane systematic review evidence supports the effectiveness of preventive care interventions involving elements included within the 5A’s approach in modifying the four priority risk behaviours [19–23]. A large proportion of the component studies tested interventions containing elements included within the 5A’s, provided by health care providers in a variety of health care settings, predominantly primary care settings [19–23]. Taken together with additional individual studies and non-Cochrane reviews undertaken in primary care settings specifically, the evidence for the effectiveness of preventive care is strongest for smoking cessation [24–28], and to a lesser extent alcohol overconsumption [14, 18, 27, 29–31], with accumulating evidence for inadequate nutrition [14, 18, 24, 27, 32] and physical inactivity [14, 18, 24, 33, 34]. While brief preventive care interventions appeared to have modest behaviour change impacts, and typically only a minority of those receiving an intervention may make clinically significant changes in risk behaviour, such an effect translates to significant health benefits at the population level when systematically applied to the large proportion of people that are at risk [14].

Within the primary care setting, nurses and allied health clinicians have the potential to be key providers of preventive care [12, 14, 17, 18, 35–39] as their care focuses on chronic disease prevention and management [12, 36], often delivered on multiple occasions to population groups with a high prevalence of behavioural risks [12, 36, 40, 41].

Despite the potential of primary care nurses and allied health clinicians to provide preventive care, variable levels of its provision have consistently been reported internationally in primary care practices [42–52]. For example, in a study in the UK, 30–50 % of primary care nurses reported they actively addressed smoking, inadequate nutrition, alcohol overconsumption, or physical inactivity with a large proportion of their clients [53]. An Australian study using client report found generalist community nurses and allied health clinicians provided brief advice for these four behavioural risks to between 43 % and 66 % of clients at risk [39]. A further Australian study [54] based on client self-report of care provision by nursing and allied health clinicians (which encompassed psychologists/psychiatrists/counsellors, social workers, occupational therapists, physiotherapists, and dieticians/nutritionists, among others) [55] found the prevalence of clinician assessment to not exceed 60 % for any of four behavioural risks; only 16 % of clients were assessed for all four risks; and referral/follow-up was offered to less than 5 % of clients for individual risks and to less than 1 % for all four risks combined [54]. Such data suggests there is a need to increase the delivery of preventive care by primary care nurses and allied health clinicians.

Cochrane reviews have examined the effectiveness of practice change interventions in improving the delivery of health care practices generally (including preventive care delivery, test ordering/utilisation, prescribing, management of a presenting problem, data recording, and diagnosis), delivered primarily by physicians in settings that included primary care [56–61]. All such reviews found practice change intervention strategies were effective in producing small to moderate improvements in the delivery of the specified health care practice. The reviews focused on the impact of the following practice change strategies: educational meetings [56], educational outreach visits and academic detailing [61], professional, financial and organisational interventions [57], audit and feedback [58], printed educational materials [59], and financial incentives [60]. Conclusions regarding the effectiveness of the utilisation of multiple of the above intervention strategies are limited by each review examining one particular type of intervention strategy.

Three systematic reviews have examined the effect of practice change strategies on delivery of preventive care for smoking [62], or alcohol consumption [63, 64] within primary care settings, with the clinicians targeted in the included studies being predominantly primary care physicians. The review on smoking care supported the effectiveness of single strategy interventions (including performance feedback, reminders and prompts, academic detailing) on some elements of care. However, it found multi-strategic interventions, defined as interventions combining two or more intervention strategies [62], to be more consistently effective [62]. The alcohol reviews also demonstrated the effect of practice change interventions: one review concluded that both educational and office-based interventions could be effective, resulting in an absolute increase of between 8 % and 18 %, with interventions that combine both strategies being most effective [63], while the second review found that alcohol screening and counselling increased with the amount of clinician training and/or support provided; however, the overall effectiveness was modest [64].

No systematic reviews could be located that examined the effectiveness of practice change strategies in increasing the delivery of preventive care specifically by primary care nurses and/or allied health professionals regarding any of four behavioural risks.

### Objectives

Given no systematic reviews have examined the effectiveness of practice change interventions in increasing primary care nurses and/or allied health professionals provision of recommended elements of preventive care for any of the four priority behavioural risks, a systematic review following PRISMA guidelines was undertaken that aimed to summarise such evidence. The current review included controlled intervention trials conducted in a primary care setting that assessed the effect of single or multi-strategic practice change interventions on preventive care provision by nurses and/or allied health professionals. Outcomes of interest were the provision of any of the five recommended elements of preventive care (ask, assess, advise, assist, or arrange) [17] for at least one of four behavioural risks (smoking, inadequate nutrition, alcohol overconsumption, or physical inactivity). Preventive care outcomes for each of the five care elements for the four risks were summarised for control and intervention groups.

## Methods

### Eligibility criteria

#### Information sources and search strategy

A search of Medline, Embase, PsycInfo, and CINAHL databases was undertaken using the following MeSH headings: (‘Primary Health Care’ or ‘Community Health Centers’ or ‘Community Health Services’ or ‘Community Health Nursing’; or ‘Attitude of Health Personnel’); and (‘Smoking’ or ‘Smoking Cessation’ or ‘Alcoholism’ or ‘Exercise’ or ‘Diet’ or ‘Preventive Health Services/og [Organization & Administration]’ or ‘Risk Factors’). The search was limited to articles published in the last 20 years, from January 1992 to 2012, and subsequently updated to be current as at May 2014.

#### Trial selection

All titles and abstracts retrieved by electronic searching were downloaded into a reference management database (Reference Manager v12), screened by the first author, and studies not meeting the inclusion criteria were excluded. Where not possible to exclude articles based on title and abstract, full text versions were obtained and their eligibility was assessed by the first author. The reference lists of included trials were checked for further relevant trials.

#### Inclusion criteria

Identified articles were examined to determine whether the following inclusion criteria were met. Each paper was assessed starting from the first criterion onwards and recorded as excluded on the first criterion it did not meet. Once excluded, the paper was not assessed against subsequent inclusion criteria.

1.  It was in English.
2.  It was a journal article (excluded grey literature such as transcribed interviews, case studies, commentaries, thesis dissertations, reflections, conference abstracts/posters).
3.  It was not a study protocol, review, or editorial.
4.  It quantitatively described at least one of five preventive care outcomes (ask, assess, advise, assist, or arrange) for at least one of the four risks (smoking, inadequate nutrition [including inadequate fruit and vegetable consumption], alcohol overconsumption, or physical inactivity). In an attempt for the search to be more inclusive, 5A’s terminology was not required and could be inferred by the extractor based on definitions of the 5A’s [17, 18]. Operational definitions of risk behaviours were not pre-specified but rather were dependent on how each trial defined such risk behaviours. Preventive care outcomes could include measures relating to clients receiving care and/or health professionals providing care.
5.  The preventive care targeted clients who were adults 18 years and older, or the citation reported care data for adults separately to children.
6.  It was in a primary care setting (including general practice, community health services, Health Maintenance Organisations, Primary Care Trusts, mobile nursing services, medical centre outpatient, university clinic, and dentistry settings). Studies excluded were those set in inpatient and outpatient hospital settings, emergency departments, and residency clinics.
7.  Preventive care was provided by routine staff members as part of routine primary care delivery, not by staff specifically employed to implement preventive care as part of the research (e.g. research assistants).
8.  It was an intervention trial that tested the effect of any practice change strategies (single or multi-strategic interventions) on preventive care provision outcome/s, and that included comparison with a control group (including controlled trials, time series, or controlled before-after trials).
9.  It included nurses or allied health professionals as the practice change intervention target. Allied health professionals included any person involved in the delivery of care (professional or not, regulated or not) that was not a nurse, midwife, or physician.
10.    It reported preventive care outcome data for nurses or allied health professionals. If other types of clinicians were involved (e.g. general practitioners, doctors, residents), the results specifically for the nurses or allied health professionals were available.

### Data extraction and description of trials

Data extraction was undertaken by the first author and recorded into a form which had been developed prior to the search and piloted. Accuracy of extraction was confirmed by a second author checking the data extraction of all variables and studies. Selected trials were summarised alphabetically and described in terms of the following: author, year published, country undertaken in, trial design, trial risk-factor focus, care setting, sample size, practice change intervention strategies utilised, clinician target group, data collection tool, preventive care practices examined, and outcome measures. A narrative synthesis was utilised. A meta-analysis was not planned as it was anticipated that studies would be too heterogeneous to provide a meaningful summary in relation to participants (e.g. clinicians or clients), interventions, and outcomes (e.g. the various health risk behaviours and care elements examined and the potential for multiple analyses to be conducted for each care element within each risk behaviour) [65].

#### Practice change intervention strategies utilised

Intervention strategy classification was based on definitions outlined by the Cochrane Effective Practice and Organisation of Care Group (Table 1). All strategies included in intervention and control group conditions were listed for each trial.

**Table 1:** Intervention strategies to change health professional practice^a^

Interventions	Definition
Distribution of educational materials	Published or printed recommendations for clinical care including clinical practice change guidelines, delivered personally or through mass mailings.
Educational meetings	Health care providers who have participated in conferences, lectures, workshops, or traineeships.
Local consensus processes	Inclusion of participating providers in discussion to ensure that they agreed that the chosen clinical problem was important and the approach to managing the problem was appropriate.
Educational outreach visits and academic detailing	Use of a trained person who met with providers in their practice settings to give information with the intent of changing the provider’s practice. The information given may have included feedback on the performance of the provider(s).
Local opinion leaders	Use of providers nominated by their colleagues as ‘educationally influential’.
Patient mediated interventions	New clinical information (not previously available) collected directly from patients and given to the provider.
Audit and feedback	Any summary of clinical performance of health care over a specified period of time. The summary may also have included recommendations for clinical action. The information may have been obtained from medical records, computerised databases, or observations from patients.
Reminders	Patient or encounter specific information, provided verbally or on paper, or on a computer screen, which is designed or intended to prompt a health professional to recall information, including computer-aided decision support.
Marketing	A survey of targeted providers to identify barriers to change and subsequent design of an intervention that addresses identified barriers.
Professional	Individual behaviour (distributing educational materials) and organisational interventions (local consensus processes).
Financial	Includes individual and organisational incentives and environmental restructuring (changing the available products).
Organisational	Includes input (changing skill mix), processes (communication), and effects (satisfaction of providers). Influencing the organisation of services, including the process of care (delegation of tasks), the structure of care (the follow-up system), and the content of care (health charts, flow sheets).
Regulatory	Includes legal (changes in patient liability) and social influence (peer review).
Patient resources^b^	Distribution or addition of resources that may aid discussions of risk factors, or allow previously unavailable options for preventive care, including flipcharts, educational resources for patients, and referral opportunities (e.g. quitlines).
Ongoing support^b^	Email, telephone, or face-to-face communications which provided support and advice, responded to questions, or problems.

^a^Modified Cochrane Effective Practice and Organisation of Care group taxonomy of professional quality improvement strategies [82]

^b^Intervention strategies not covered by EPOC criteria

#### Risk of bias

To provide an indication of the methodological quality of studies, risk of bias was independently assessed by three review authors (at the outcome level of relevance to the current review; KM, KB, and PW) using the tool outlined in the Cochrane Handbook for Systematic Reviews of Interventions (Additional file 1) [65]. Discrepancies were resolved by consensus among all reviewers and a fourth review author (MF). Sources of bias assessed were those attributable to generation of the random sequence, allocation concealment, blinding of participants and personnel, blinding of outcome assessors, completeness of outcome data, selective reporting, and any other potential threats to validity. Trial authors were contacted via email (including a follow-up email to non-responders) to obtain further information regarding unclear classifications. Results were described narratively.

### Practice change intervention effect on preventive care delivery

The following outcome data for each trial were summarised: clinician provision (prevalence, means and standard deviations, and Likert scores) of any of the five elements of preventive care (ask, assess, advise, assist, or arrange) [17] with regard to the four behavioural risks (smoking, inadequate nutrition, alcohol overconsumption, or physical inactivity). All types of outcome data were summarised (e.g. clinician or client self-report, medical records audit, observations). Follow-up levels of care for control and intervention groups for each trial were described (baseline levels were reported when available), along with results of significance testing.

## Results

### Trial selection

**Fig. 1:**
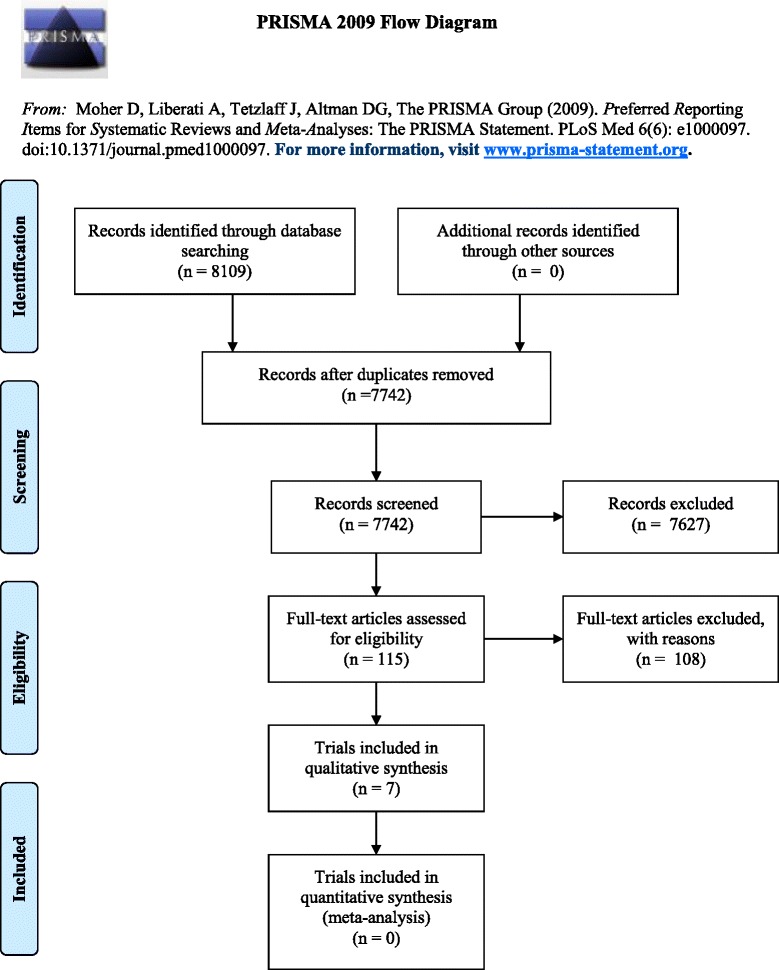
PRISMA 2009 flow diagram

In total, 8109 citations were extracted from the search (see Fig. 1 for PRISMA flow diagram). After duplicates were removed (*n* = 367), 7742 abstracts and titles were reviewed. Of these, 7735 articles were excluded as the following: two were not in English; 18 were not journal articles; 7189 were a study protocol, review, or editorial, or did not quantitatively describe the proportion of health professionals providing, or clients provided with preventive care for at least one of the four risks; 25 were based on data related to care for children; 102 were not in a primary care setting; 16 described care that was not provided by routine staff; 308 were not intervention trials or did not have a control/comparison group; 52 did not include nurses or allied health professionals as a clinical target of the practice change intervention; and 23 did not report results for nurses or allied health professionals separately from other types of clinicians (e.g. medical practitioners). Consequently, seven trials were included in the current review (Table 2) [66–71]. No additional trials were identified from reference lists.

**Table 2:** Intervention trials reporting change in routine provision of preventive care: January 1992–May 2014

Author/year/country/trial design	Trial focus/care setting/sample size	Intervention strategies	Clinician group/data collection tool	Preventive care practices examined/outcome
-Bakker et al. (2003) [66] -Netherlands -Cluster RCT	-Smoking cessation for pregnant women. -42 pre-natal care clinics (22 IV, 20 C) 118 midwives: -IV *n* = 57 (37 did questionnaire-65 %) -C *n* = 61 (32 did questionnaire-52 %) 556 clients: -IV *n* = 253 (sample for presented analysis = 44) -C *n* = 303 (sample for presented analysis = 51)	IV: -Distribution of educational -Offered educational meetings -Patient resources C: -Patient resources	Clinician target: -Midwives -Clinician questionnaire at follow-up. Client questionnaires. [M (SD), C vs IV] -Scale 1–5 for clinician self-report data (1 = never, 5 = always). -Scale 0–1 for client self-report (0 = no, 1 = yes). Continuous variables.	Ask: -Clinician: 5.00 vs 4.91 (0.37)^a^ -Client: 0.72 (0.29) vs 0.91 (0.18)** Advise: (to quit) -Clinician: (during pregnancy): 4.19 (1.03) vs 4.60 (0.77)* (to partner): 3.58 (1.42) vs 4.03 (1.33)^a^ -Client (during pregnancy): 0.64 (0.36) vs 0.85 (0.25)** (post-partum): 0.04 (0.08) vs 0.21 (0.25)** Assist: -clinician: 1.63 (1.10) vs 3.63 (1.19)*** -client: 0.03 (0.16) vs 0.33 (0.34)*** Arrange: -clinician: 2.84 (0.99) vs 3.97 (0.89)*** Results similar when analysed at practice level (except clinician reported advice to quit during pregnancy no longer statistically significant).
-Chan et al. (2013) [67] -Australia -Quasi-experimental design	-Relevant risks: Smoking, nutrition, alcohol consumption, and physical inactivity Other risks: weight -4 generalist community nursing services randomised to: -IV: *n* = NR -C: *n* = NR Clinicians: -Baseline: *n* = 129/178 (72.5 %) -6 months F/U: *n* = 81/129 (62.8 %) -12 months F/U: *n* = 65/129 (50.4 %) Overall response rate across all time points *n* = 54/129 (41.9 %)	IV (early IV): -Educational meetings -Reminders -Patient resources -Distribution of educational materials C (late IV): -Usual care -Followed by intervention after collection of outcome data	Clinician target: -Generalist community health nurses -Clinician questionnaire: -Baseline -6 months F/U -12 months F/U [Group mean effect size (95 % CI) at 6 and 12 months F/U, respectively. Based on Likert scale 1 (never)-7 (always) provided care as part of routine practice].^e^ [For ask and advise/assist scores: tested for significant differential change between IV vs C groups over time (baseline, 6 and 12 mths F/U). (Time by group interaction p value). For Arrange scores: significance testing conducted for IV vs C groups at baseline vs 6 mths, and baseline vs 12 mths]	Ask: -S: 0.15 (−0.40–0.69), 0.30 (−0.26–0.84)^a^ -N: 0.26 (−0.29–0.80), 1.12 (0.52–1.69)*** -A: 0.60 (0.01–1.16), 0.56 (−0.02–1.13)^a^ -P: 0.34 (−0.21–0.89), 0.72 (0.15–1.27)*** Advise/assist -S: 0.48 (−0.12–1.06), 0.42 (−0.17–1.00)*** -N: 0.09 (−0.46–0.64), 0.30 (−0.26–0.85)^a^ -A: 0.23 (−0.33–0.79), 0.31 (−0.26–0.86)^a^ -P: 0.36 (−0.21–0.92), 0.05 (−0.51–0.60)** Arrange: -S (Quitline): 0.17 (−0.38–0.71),^a^ 0.39 (−0.16-0.93)^a^ -N: 0.09 (−0.45–0.63),^a^ 0.10 (−0.45–0.64)^a^ -A: −0.39 (−0.93–0.16),^a^ −0.13 (−0.67–0.42)^a^ -P: −0.33 (−0.87–0.22),^a^ −0.29 (−0.83–0.26)^a^
-Kaner et al., (2003) [68] -UK -Cluster RCT	-Alcohol -212 general practices randomised to: -C: *n* = 76 -IV 1: *n* = 68 -IV 2: *n* = 68 (data for 156 practices)	IV 1—Outreach Training -Educational outreach visit -Patient mediated intervention -Educational meeting IV 2—Training plus telephone-based support -Educational outreach visit -Patient mediated intervention -Educational meeting -Ongoing support C: -Patient mediated intervention -Distribution of educational materials	Clinician target: -Nurses Clinician questionnaire: -Baseline -3 months F/U (collection of screening forms) (Median [interquartile range], C vs IV 1 vs IV 2)	Ask: 0 [0–17] vs 11 [0–28] vs 13 [0–37]** Advise: 0 [0–3] vs 1 [0–4] vs 1 [0–7]* (% of clients) 60 vs 61 vs 64 %*
-Katz et al. (2004) [69] -USA -RCT	-Smoking cessation -9 primary care clinics (routine non-emergency care: 7 family practice, 2 internal medicine) -Patients: *n* = 1221 (includes patients seen by medical assistants) *n* = 663 patients seen by below clinicians: IV sites: -Registered nurses: *n* = 100 -Licenced practical nurses: *n* = 154 C sites: -Registered nurses: *n* = 153 -Licenced practical nurses: *n* = 256	IV (*clinician targeted*): -Educational meeting -Audit and feedback -Reminders -Patient resources C: -Unspecified	Clinician target: -Registered nurses, -Licenced practical nurses -(Medical assistants)^e^ Client interviews (during IV period) (% C vs IV site patients receiving care)	Ask: -Registered nurses: 67 % vs 92 %^b^ -Licenced practical nurses: 35 % vs 86 %^b^ Assess: -Registered nurses: 15 % vs 85 %^b^ -Licenced practical nurses: 8 % vs 75 %^b^ Advise: -Registered nurses: 16 % vs 41 %^b^ -Licenced practical nurses: 7 % vs 46 %^b^ Assist: -Registered nurses: 17 % vs 73 %^b^ -Licensed practical nurses: 8 % vs 69 %^b^
-Lennox et al., (1998) [72] -UK -RCT	-Smoking -Primary care: 16 general practices: (IV, *n* = 8; C, *n* = 8). Clinicians receiving IV: Practice nurses: 15/16 (93.7 %) Health visitors: 16/16 (100 %) Clients: 14 months F/U response rate: 1693/2588 (65.4 %).	IV: -Educational meeting C: -Usual care (no educational meeting)	Clinician target: -Practice nurses -Health visitors -(General practitioners)^c^ Client questionnaire: -14 months F/U (% (*n*) of C vs IV patients)	Ask: Practice nurses: 76.2 % (*n* = 77/101) vs 83.2 % (*n* = 104/125)^a^ Health visitors: 68.6 % (*n* = 24/35) vs 73.7 % (*n* = 28/38)^a^
-Moher et al., (2001) [70] -UK -Cluster RCT	-Coronary heart disease Relevant risks: smoking Other risks: blood pressure, cholesterol -Primary care: 21 general practices: (IV 1, *n* = 7; IV 2, *n* = 7; C, *n* = 7). Clients: *n* = 4048 Baseline (IV 1, *n* = 772; IV 2, *n* = 747; C, *n* = 623). F/U: (IV 1, *n* = 682; IV 2, *n* = 665; C, *n* = 559).	Nurse targeted IV: -Audit and feedback -Local consensus processes -Educational outreach visits and academic detailing -Ongoing support -Patient mediated intervention C: -Audit and feedback -Usual care	Clinician target: -Nurses -(General practitioners)^c^ Medical records audit: -Baseline -18 months F/U [Mean % of clients (range), C vs general practitioners targeted IV vs nurse targeted IV]	Ask: *** -Baseline: 73 % (50–91) vs 71 % (47–96) vs 71 % (46–85) -F/U: 78 % (56–92) vs 92 % (77–100) vs 95 % (88–98)
-Secker-Walker et al., (2000) [71] -USA -Non-RCT	-Smoking in women (18–64 years) -Primary care: *n* = 4 2 IV counties 2 C counties Clinicians: *n* = 289 (eligible) -Dentists (IV: *n* = 51, C: *n* = 46), -Dental hygienists (IV: *n* = 38, C: *n* = 44), -Family planning counsellors and WIC^f^ nurse counsellors (IV: *n* =14 C: *n* =16), -Community mental health counsellors (IV: *n* = 57, C: *n* = 23) -Physicians (IV: *n* = 73, C: *n* = 73),	-IV *(4-year multi-strategic, clinician targeted)*: Family Planning and WIC nurse counsellors: -Educational meeting -Patient resources Dentists, and dental hygienists: -Educational meeting -Ongoing support Mental health counsellors: (no formal approaches, but educational meeting attended by 3 staff members) C: -Usual care	Clinician target: -dentists -dental hygienists -family planning counsellors and WIC nurse counsellors Clinician questionnaire: -Baseline -Yr 5 F/U -Yr 7 F/U (Means for baseline vs F/U % of smokers receiving cessation activity on 4 point scale: 0 = none, 1 = some, 2 = most, 3 = all). (Time by group interaction p value). Paired comparisons at Yr 5 *(Paired)* (Means for C vs IV counties % of smokers receiving cessation activity on a 4 point scale: 0 = none, 1 = some, 2 = most, 3 = all) Unpaired comparisons at Yr 5; Unpaired comparisons at Yr 7 *(Unpaired)*	*Paired Comparisons (Yr 5)* Advise (to quit): -Dentists: IV: 1.7 vs 1.7;^d^ C:1.3 vs 1.6^a^ Assist (provision of self-help materials): -Dentists: IV: 0.2 vs 0.6; C: 0.3 vs 0.3* -Dental hygienists: IV: 0.3 vs 0.7; C: 0.4 vs 0.4^a^ Arrange (Referral to support group): -Dental hygienists: IV: 0.2 vs 0.6; C: 0.1 vs 0.1;** Arrange (Referral to quit group): -Dentists: IV: 0.1 vs 0.4; C:0.2 vs 0.2;* -Dental hygienists: IV: 0.3 vs 0.9; C: 0.3 vs 0.4;** *Unpaired Comparisons (Yr 5 and Yr 7)* Assist (provision of self-help materials): -Family Planning and WIC counsellors: Yr 5: 1.1 vs 1.7;** Yr 7: 1.5 vs 1.6^a^ Assist (setting quit dates): -Family planning and WIC counsellors: Yr 5: 0.4 vs 0.8;* Yr 7: 0.7 vs 0.6^a^ Arrange (referral to support group): -Dentists: Yr 5: 0.0 vs 0.2;* Yr 7: 0.2 vs 0.2^a^ -Dental hygienists: Yr 5: 0.1 vs 0.6;*** Yr 7: 0.2 vs 0.4^a^ -Family planning and WIC counsellors: Yr 5: 0.4 vs 1.3;*** Yr 7: 0.3 vs 0.3^a^ -Community mental health counsellors: Yr 5: 0.1 vs 0.4;* Yr 7: 0.3 vs 0.3^a^ Arrange (referral to quit group): -Dentists: Yr 5: 0.2 vs 0.4;* Yr 7: 0.3 vs 0.4^a^ -Dental hygienists: Yr 5: 0.4 vs 0.9;** Yr 7: 0.3 vs 0.5^a^ -Family planning and WIC counsellors: Yr 5: 0.7 vs 1.7;*** Yr 7: 1.0 vs 0.6^a^ Arrange (referral one-to-one telephone support): -Family planning and WIC counsellors: Yr 5: 0.3 vs 1.4;*** Yr 7: 0.5 vs 0.6^a^

*p ≤ 0.05, **p ≤ 0.01, ***p ≤ 0.001

*RCT* randomised control trial, *IV* intervention, *C* control, *NR* not reported, *F/U* follow-up, *S* smoking, *N* nutrition, *A* alcohol, *P* physical activity, *NR* not reported

^a^Not statistically significant at *p* < 0.05

^b^Significance testing not conducted

^c^Paper reported results separately for this clinician target

^d^Correctly reported from paper. Confidence intervals (1.3–2.0 vs 1.4–2.0)

^e^Note: only effect size results summarised. Group M and 95 % CI for IV and C groups at baseline, 6- and 12-month follow-up reported but not summarised in table due to space constraints

^f^WIC refers to Special Supplemental Food Program for Women, Infants and Children

### Trial characteristics

#### Year, country, and trial design

All seven trials were published between 1998 and 2013 (one later than 2004); two were undertaken in the USA [69, 71], three in the UK [68, 70, 72], one in Australia [67], and one in the Netherlands [66]. Four trials were randomised controlled trials [68–70, 72]; one was a cluster randomised controlled trial [66], one was a non-randomised controlled trial [71], and one was a quasi-experimental design [67].

#### Primary care setting and sample size

Trials were conducted in primary care practices/clinics/general practices [68–72], prenatal care clinics [66], and generalist community nursing services [67]. The number of practices ranged from 4 to 212 [66–70, 72]. For the four trials reporting sample sizes for clients, these ranged from 556 to 4048 clients [66, 69, 70, 72]. For the three trials reporting sample sizes for clinicians involved in outcome assessment, these ranged from 30 to 129 nurses [66, 67, 71] and 80 to 97 allied health clinicians [71].

#### Intervention strategies

One trial used one practice change intervention strategy [72], two trials used three strategies [66, 71], three used four strategies [67–69], and one used five [70]. Six of the seven trials reported utilising educational meetings as an intervention strategy [66–69, 71, 72], four reported using patient resources [66, 67, 69, 71], and each of the following strategies was reported to be utilised by two trials: audit and feedback [69, 70]; patient-mediated intervention [68, 70]; educational outreach visits and academic detailing [68, 70]; ongoing support [68, 70]; distribution of educational materials [66, 67]; and reminders [67, 69]. One trial used local consensus processes [70]. The control condition consisted of usual care for three trials [67, 71, 72], minimal intervention strategies in three (e.g. patient resources [66], audit and feedback [70], and patient-mediated intervention [68] and distribution of educational materials [68]), and was not specified in one trial [69].

#### Clinician group receiving the intervention and data collection tools

The nursing or allied health clinician groups that were the target of the practice change intervention for these trials were predominantly nurses (7/7 trials, including general practice nurses [68, 70, 72], generalist community health nurses [67], registered nurses and licenced practical nurses [69], midwives [66], health visitors [72], and family planning counsellors, and WIC nurses counsellors [71]). Only one trial included allied health professionals (in addition to nurses) including dentists, dental hygienists, and mental health counsellors [71].

Clinician questionnaires were used to measure preventive care delivery in three trials [67, 68, 71], with medical records audit [70], client questionnaires [72], client interviews [69], and a combination of client and clinician questionnaires also used [66].

#### Behavioural risks addressed

One trial focused on all four of the behavioural risks [67], with all others focusing on only one of these risks (five on smoking [66, 69–72] and one on alcohol overconsumption [68]).

#### Preventive care practices

The trial reporting preventive care provision regarding all four risks utilised a reduced model of care that focused on risk assessment, brief advice/assistance, and referral [67]. The five trials that focused on smoking only examined risk assessment (4/5) [66, 69, 70, 72], assessment of readiness to change (1/5) [69], advice (4/5) [66, 69–71], assistance (4/5) [66, 69–71], and arranging referral (3/5) [66, 70, 71]. The one trial that focused on only alcohol overconsumption examined risk assessment and advice [68]. For trials that addressed ‘arranging referral’, the variables of interest were referral to other service providers/support services for each of the four risks (including a quitline for smoking) in the multiple-risk trial [67], and referral to the following in the smoking-focused trials; a support group [71], a quit group [71], one-to-one telephone support [71], or discussing ‘aftercare’ [66].

### Risk of bias

Table 3 provides a summary of judgements regarding the risk of bias at the outcome level for each trial. Overall, trial quality was difficult to assess given insufficient information reported regarding risk of bias classifications. Five trial authors supplied further information regarding unclear classifications upon contact [66–70]. All trials had at least one high risk of bias judgement, and as such, none were judged to be of high methodological quality.

**Table 3:** Summary of risk of bias in individual trials

Intervention trial author, year, and trial design	Random sequence generation (selection bias)	Allocation concealment (selection bias)	Blinding of participants and personnel (performance bias)	Blinding of outcome assessment (detection bias)	Incomplete outcome data (attrition bias)	Selective reporting (reporting bias)	Other potential sources of bias
Bakker -(2003) [66] -Cluster RCT, cross-sectional design (post-test only).	Low	Unclear	Unclear	Unclear^a^	Low^a^	Unclear	High
Chan -(2013) [67] -Quasi-experimental design	High	High	High	Low	High	Low	High
Kaner -(2003) [68] -Cluster RCT	Low	Low	Low	High	Low	Unclear	High
Katz -(2004) [69] -RCT	Low	Unclear	Unclear	Unclear	Low	Unclear	High
Lennox -(1998) [72] -RCT	Low	Unclear	Low	Low	Low	High	High
Moher -(2001) [70] -Cluster RCT	Low	Low	High	High	Low	Unclear	High
Secker-Walker, -(2000) [71] -Non-RCT	Unclear	Unclear	Unclear	Unclear	High	High	High

^a^Same risk of bias judgement for both classes of outcomes (clinician reported and client reported)

### Results of trials

#### Intervention effect on preventive care delivery

Of the seven trials, six conducted significance testing, including the multiple-risk trial [66–68, 70–72]. Trials reporting a significant increase in at least one variable included three [66, 71], four [67, 68], or five [70] of the following interventions strategies (from most to least frequent): educational meetings [66–68, 71], patient resources [66, 67, 71], patient-mediated intervention [68, 70], educational outreach visits and academic detailing [68, 70], ongoing support [68, 70], distribution of educational materials [66, 67], audit and feedback [70], reminders [67], and local consensus processes [70].

##### Smoking

Of the four trials that sought to enhance the delivery of smoking assessment (‘ask’) [66, 67, 70, 72], two showed a positive effect for at least one measure of clinician assessment of client risk [66, 70] (the multi-risk trial found no effect). Of the three trials examining smoking brief advice [66, 67, 71] (one examining a combined measure of brief advice and assistance) [67], two showed a positive effect of the intervention for at least one measure of brief advice [66, 67]. Of the two trials examining smoking assistance (specifically the provision of self-help materials [71] and setting quit dates [66, 71]), both demonstrated a positive effect of the intervention for at least one measure of assistance [66, 71]. Lastly, of the three trials that sought to enhance arranging referral for smoking [66, 67, 71], two demonstrated a positive effect of the intervention for at least one measure of the provision of referral (specifically discussing aftercare [66] and referral to a support group, quit group, and telephone support [71]). However the multi-risk trial found no effect for smoking referral [67].

The studies reported multiple analyses for each care element, for example using different variables [66, 71], conducting different analyses for different data collection methods [66], or clinician subgroups [71, 72], using different statistical techniques [71], or examining different follow-up points [67, 71]. Of the six analyses of smoking risk assessment conducted in four trials [66, 67, 70, 72], two demonstrated positive intervention effects [66, 70]. Of the 15 brief advice analyses conducted in three trials [66, 67, 71], four demonstrated positive intervention effects [66, 67]. Of the 22 smoking assistance analyses conducted in two trials [66, 71], five demonstrated positive intervention effects [66, 71]. Of the 39 smoking referral analyses conducted in three trials [66, 67, 71], nine demonstrated positive intervention effects [66, 71].

##### Alcohol

The following results were found for the two trials with a focus on alcohol preventive care provision [67, 68]. Both trials included a measure of alcohol risk assessment and brief advice measures [67, 68]; one trial examined a combined measure of brief advice and assistance) [67] and only one trial showed a positive effect of the intervention on assessment and advice [68]. The trial examining all four risks simultaneously found no effect with regard to alcohol assessment or brief advice [67]. In the one trial examining arranging alcohol referral (the multi-risk trial), the intervention effect was not significant [67].

##### Inadequate nutrition and physical inactivity

No trials focused solely on increasing care for inadequate nutrition and physical inactivity as independent risks. However, with regard to nutrition risk assessment, the multiple-risk trial demonstrated a positive effect of the intervention. With regard to nutrition brief advice and arranging referral, neither measure showed a significant intervention effect [67]. With regard to physical inactivity assessment and brief advice, this trial demonstrated a positive effect of the intervention [67]. However, with regard to arranging physical inactivity referral, there was no significant intervention effect [67].

## Discussion

Seven intervention trials were located that described the effectiveness of strategies to increase the provision of preventive care regarding smoking, inadequate nutrition, alcohol overconsumption, or physical inactivity by primary care nurses or allied health clinicians. The trials were predominantly undertaken over 10 years ago (6/7), and only one included professionals other than nurses. While there was some evidence to indicate that practice change interventions for such clinicians may be effective in increasing the provision of smoking cessation care, this was limited given the small number of studies and the inconsistency of effect between and within trials. The effectiveness of interventions to increase care for alcohol overconsumption, inadequate nutrition, and physical inactivity and for multiple risks is unclear given the very small number of trials that examined care regarding these risk factors. Such conclusions are further qualified as no trials were judged to be of high methodological quality. Additional research is needed to determine the capacity of interventions to increase the provision of multiple elements of preventive care for these four priority behavioural risks by both nurses and allied health professionals in primary care settings.

The suggestion that interventions may be effective for smoking cessation care is based on the significant increase in at least one preventive care element reported in four of the five included trials that examined smoking and conducted significance testing. For the one trial that reported an effect size for smoking cessation care (17 %) [70], the effect size was consistent with past Cochrane reviews examining the effect of practice change strategies on health care practices more broadly [56–61]. In such Cochrane reviews, small to moderate improvements in care delivery were noted, with median adjusted [56, 58, 61] or absolute [59] effect size differences ranging from 2 % [59] to 6 % [56] for categorical professional outcomes [56, 58, 59, 61] and from 1.3 % [58] to 21 % [61] for continuous outcomes [56, 58, 59, 61]. However, in the current review, the lack of consistency of the effectiveness of the multi-strategic approach within and between studies necessitates that conclusions regarding effectiveness on smoking care are made cautiously. When summaries are based on number of analyses undertaken for each care element, the results are less positive. For example, of the 39 smoking referral analyses conducted in three trials [66, 67, 71], only seven demonstrated a positive effect of the intervention [66, 71]. We would conclude that the results are unclear.

Further, conclusions with regard to alcohol overconsumption, nutrition, and physical inactivity cannot be drawn. Only two trials examined preventive care regarding alcohol overconsumption. Such trials reported conflicting findings and suggested relatively small or no effects [67, 68]. This differed to the modest effectiveness reported in the aforementioned systematic reviews based on a larger number of trials (11–12) that examined effect of practice change on delivery of preventive care for alcohol within primary care settings (care predominantly by physicians) [63, 64]. With regard to increasing inadequate nutrition and physical inactivity, only one trial was identified and reported variable effect by risk and care element.

The findings indicate the need for further investigation of intervention approaches that may result in an increased prevalence of care across risk factors and care elements [73]. All trials in the current review, bar one [72], used a multi-strategic intervention including between three and five practice change strategies. While such an approach is supported by other studies and reviews recommending the inclusion of multiple practice change strategies within intervention trials [38, 62–64, 74–79], an overview of systematic reviews evaluating the effectiveness of multi-strategic interventions in changing health care professional’s behaviour in clinical settings found no compelling evidence that such interventions are more effective than single strategy interventions [80]. Strategies implemented might be informed by barriers to care delivery at the client, clinician, and system level in light of review evidence supporting the effectiveness of tailoring intervention strategies to determinants of practice (barriers, obstacles, enablers, and facilitators) [81]. In regard to smoking cessation care specifically, a multi-strategic approach is recommended by various clinical guidelines [1, 7, 38]. For example, the United States Treating Tobacco Use and Dependence Clinical Practice Guidelines recommend the implementation of a tobacco user identification system; provision of education, resources, and feedback to promote provider intervention; dedicate staff to provide tobacco dependence treatment, and assess its delivery in staff performance evaluations; promotion of hospital policies that support and provide inpatient tobacco dependence services; and the inclusion of tobacco dependence treatments as paid or covered services in all subscribers or members of health insurance packages [38].

The findings of this review should be considered in light of a number of limitations. The generalisability of the review conclusions to allied health clinicians is limited as only one trial included allied health clinicians, and this trial also included nurses. Similarly, generalisability across primary care settings is limited as most included trials were conducted in a limited range of settings, predominantly primary care practices/clinics/general practices [68–72], with few or no studies in settings such as community health services, Health Maintenance Organisations, Primary Care Trusts, or mobile nursing services. Additionally, the trial results are predominantly from studies published over 10 years ago and hence more current evidence regarding the effectiveness of practice change interventions for primary care nurses and allied health professionals is unclear. Furthermore, as a consequence of inferring 5A’s terminology for studies included in the review, definitions of what constituted the same element of care could vary and hence may account for some of the variability between and within studies on such measures. Also, although the current review utilised a broad search strategy, only trials published in journals within the included databases were located [65]. Finally, the search was also limited by having only one author conducting the title and abstract review.

### Conclusions

The current review indicated that there is little evidence on the effectiveness of practice change interventions for primary care nurses and allied health professionals. The small number of trials focused on care for smoking shows intervention effects to be inconsistent between and within studies. Evidence for the effectiveness of interventions to increase care for alcohol overconsumption, inadequate nutrition, and physical inactivity and for multiple risks is also inconclusive as they were examined in an even more limited number of trials with inconsistent findings. There is a need for further research with regard to effective interventions to increase preventive care by nurses and in particular allied health professionals in primary care settings. Such research could examine a range of care elements regarding smoking, alcohol overconsumption, inadequate nutrition, physical inactivity, and for multiple risks.

## Additional file

Additional file 1: Table S1. The Cochrane Collaboration’s tool for assessing risk of bias [65]. **Table S2.** Criteria for judging risk of bias in the ‘Risk of bias’ assessment tool [65]. (DOCX 53 kb)
